# Reliability and validity of Japanese versions of the UCLA loneliness scale version 3 for use among mothers with infants and toddlers: a cross-sectional study

**DOI:** 10.1186/s12905-019-0792-4

**Published:** 2019-07-26

**Authors:** Azusa Arimoto, Etsuko Tadaka

**Affiliations:** Department of Community Health Nursing, Division of Nursing, Graduate School of Medicine, Yokohama City University, 3-9 Fukuura, Kanazawa-ku, Yokohama, Japan

**Keywords:** Assessment, Infant, Instrument development, Loneliness, Maternal and child health, Mothers, Public health nurse

## Abstract

**Background:**

Mothers with infants and toddlers are a potential target population for the prevention or alleviation of feelings of loneliness. However, the theory and methods for measuring loneliness among mothers with infants and toddlers have yet to be standardized worldwide, including in Japan. Our goal was to evaluate the reliability and validity of the Japanese version of the UCLA Loneliness Scale Version 3 (UCLA-LS3-J), as well as two short-form versions—the 10-item UCLA-LS3 (SF-10) and the 3-item UCLA-LS3 (SF-3)—for the measurement of loneliness in mothers with infants and toddlers in Japan.

**Methods:**

This cross-sectional study was conducted using a self-report questionnaire. The target population was 430 mothers with infants and toddlers who visited a community health center in Yokohama City in Japan. Questionnaire items encompassed the UCLA-LS3-J, as well as demographic data, the feeling for childrearing scale, and measures of social networks and subjective health status. The reliability and validity of the UCLA-LS3-J and its two short-form versions (SF-3 and SF-10) were determined via IBM SPSS Amos and SPSS Statistics 22.

**Results:**

Questionnaires were returned by 248 mothers (valid response rate: 57.7%) aged 32.7 ± 4.6 (mean ± SD) years. The mean score on the UCLA-LS3-J was 38.4 ± 9.7 (range 20.0–73.0), with a normal distribution. When confirmatory factor analysis was carried out (for a single factor model), the goodness of fit of the model was almost identical to that of the original UCLA-LS3 version for the UCLA-LS3-J: (GFI = 0.882, AGFI = 0.840, CFI = 0.932, RMSEA = 0.066) and SF-10: (GFI = 0.942, AGFI = 0.900, CFI = 0.956, RMSEA = 0.081). The SF-3 model also showed an acceptable fit. The UCLA-LS3-J total score was significantly correlated with the total score on the SF-10 (r = 0.965) and SF-3 (r = 0.868). The Cronbach’s α coefficient of the UCLA-LS3-J was 0.926, while those of the SF-10 and SF-3 were 0.888 and 0.790, respectively. The score on the UCLA-LS3-J was positively correlated with childcare burden (r = .319, *p* < 0.001) and negatively correlated with social networks (r = −.438, p < 0.001).

**Conclusions:**

This study indicated that the reliability and validity of the UCLA-LS3-J as well as its two short-form versions were adequate for assessing loneliness in mothers with infants and toddlers in Japan.

**Electronic supplementary material:**

The online version of this article (10.1186/s12905-019-0792-4) contains supplementary material, which is available to authorized users.

## Background

### Loneliness and health

Loneliness is “the unpleasant experience that occurs when a person’s network of social relations is deficient in some important way, either quantitatively or qualitatively” [1]. Loneliness is one of the most important issues that regional communities currently face. Previous studies have reported that the prevalence of experiencing loneliness at some time in one’s life is 80% among adolescents and 40% in elderly over 65 years old [2–4]. Obtaining precise estimates for the prevalence of loneliness and social isolation is difficult due to variation across the life course, cultural and gender differences with respect to individuals’ readiness to talk about themselves from a personal perspective, and the use of various measurement scales, some of which are based on self-report questionnaires and others that involve more objective assessment of social contact or networks [5]. Pinquart and Sorenson [3] showed that the degree of loneliness gradually decreased thorough middle adulthood and increased in individuals over 70 years old. However, loneliness remains chronic among approximately 15–30% of the general population [6, 7].

Loneliness is recognized as an important factor in various healthcare issues. For instance, it is closely related to issues such as youth mental health [8], childhood abuse [9], mature age alcohol dependency [10], and depression [11]. On closer examination, it is clear that loneliness is related to a wide range of health risk factors among mothers and young to middle-aged adult women, such as life satisfaction [12], subjective perception of health [13], depression [11], onset of heart disease [14], and increased risk of death [15]. Chronic feelings of loneliness are related to a decline in mental well-being and to symptoms such as anxiety and depression [6, 16, 17].

Social isolation is distinguished from loneliness because social isolation is when more structural and rather objective characteristics of social relationships cover the number and type of people with whom a person interacts, the diversity, density and reciprocity of a person’s social network, and frequency and duration of contact between individuals [18]. Reviews on scales measuring social relationships reported that social isolation is usually characterized as an objective lack of meaningful and sustained communication, while loneliness is referred to as the way people perceive and experience the lack of interaction [19]. Isolated from the community, mothers with infants and toddlers often feel a high degree of loneliness and lower psychological well-being, which typically manifest as depression [17, 20] or childrearing anxiety [17, 21]. Maternal depression and childrearing anxiety have been acknowledged as one of the major social issues that Japan faces [21, 22]. Mothers in Asia have more responsibility for childcare compared to mothers in Western countries [23]. Therefore, mothers with infants and toddlers are a potential target population for the prevention or alleviation of feelings of loneliness. However, reports on the subjective state of isolation and loneliness among mothers are limited and have not used standardized questionnaires or measurement tools with adequate reliability and validity. To prevent lower psychological well-being among mothers with infants and toddlers, and obtain more subjective reports of isolation, it is essential to conduct and evaluate empirical studies that focus on loneliness [24].

The theory and methods for measuring loneliness among mothers have yet to be standardized worldwide. Much previous research separates the concepts of loneliness from the subjective state of isolation, in which one has practically no contact with their family and community. Loneliness is due to a lack of social relationships, is a subjective experience, and is an uncomfortable, painful experience [25, 26]. Japan does not have a tool with established reliability and validity for measuring the loneliness in mothers, based on these parameters. Limited studies have focused on loneliness in mothers with infants and toddlers [17, 21, 22, 27–32].

While there have been several scales developed for measuring loneliness thus far [33, 34], all of them are multidimensional, lacking context, and specifically use the term “loneliness.” Adopting a multidimensional approach makes it difficult to compare loneliness among different individuals. As for the context, measures that consider loneliness as an individual attribute, rather than the result of the current circumstances or environment, tend to interpret loneliness as difficult to alter. Finally, measurement tools that use the term “loneliness” may lead to responses that are biased in a socially desirable direction. For these and other reasons, previously developed scales are problematic for research purposes.

### Current state of literature

The University of California, Los Angeles Loneliness Scale version 3 (UCLA-LS3) [35], which is a revision of the original version of UCLA-LS [36] by Russell, has been adapted and validated in various subjects in numerous different countries, including Australia [37], Turkey [38], Northern Ireland [39], Iran [40], Italy [41], and Japan [42]. These adapted scales have great feasibility and applicability in their respective populations. The scale comprises 20 items, which have consistently displayed a high level of convergent validity and internal consistency [35]. Several short-form versions of the scale have been developed as well. Specifically, a 10-item version (SF-10), based on the unidimensional UCLA-LS3, was developed in 1996 [35], and a 3-item version (SF-3), based on the 20-item multidimensional UCLA-LS revised [43], was developed by Hughes [44]. The SF-10 has been an adequate fit to the unidimensional model of the UCLA-LS3 [35]. The three items on the SF-3 were selected because they showed the highest loading on the first factor of a three-factor model [44]. A comparison study of the short-forms of the UCLA-LS [37] revealed that the SF-10 [35] and the SF-3 [44] are both reliable and valid. Therefore, the UCLA-LS3 as well as its two short-form versions (SF-10 and SF-3 have become some of the most widely used measures of loneliness worldwide.

A Japanese version of the UCLA-LS3 (UCLA-LS3-J) was developed by Masuda and Tadaka [42] using the standard procedure of scale development (to ensure its fidelity across different language versions) after the second author (ET) obtained permission to translate the UCLA-LS3-J from its original author (Dr. Russell). The UCLA-LS3-J has adequate reliability and validity for use with the elderly [42]. We hypothesized that the UCLA-LS3-J, SF-10, and SF-3 are all applicable for use with mothers with infants and toddlers for the following reasons. First, the reliability and validity of the UCLA-LS3 was originally established not only for elderly adults but also for adolescents and adults, such as nurses, students, and teachers [35]. Second, the conceptualization of loneliness proposed by the UCLA-LS3 and its two short-form versions might have a degree of universality (i.e., unaffected by culture and generation), as found in previous studies [10, 35–44]. Nevertheless, the validity and reliability of the UCLA-LS3-J and the short-form versions have not been evaluated for use with mothers with infants and toddlers.

### Aim of current study

The aim of the current study was to evaluate the reliability and validity of the UCLA-LS3-J, as well as its two short-form versions (SF-10 and SF-3), for the measurement of loneliness in mothers with infants and toddlers in Japan.

## Methods

### Design

This cross-sectional study was conducted in Japan between September and November 2012.

### Participants and setting

The target population was mothers with infants and toddlers who visited a community health center for their child’s medical health-check up in Yokohama City, which is the second largest city in Japan, in 2012. Health check-ups, including growth and development examinations and health counseling, are mandatory at 4 and 18 months of age under the Maternal and Child Health Act in Japan. The sample was mothers of 4- and 18-month-old infants, for a total sample size of 430 mothers. The desired sample size was set at one quarter of 2,000 (500), based on the number of births per year in this district. Mothers who could understand Japanese and answer questionnaire items were eligible for participation. All eligible mothers were asked to complete questionnaires and return them by mail. Exclusion criteria were mothers who could not understand Japanese and answered less than half of the questionnaire items.

### Procedures

The questionnaire was sent by mail to 430 mothers along with reminder letters for a health check-up. Of these, 248 mothers (valid response rate: 57.7%) responded with questionnaires fully completed with valid responses.

### Measures

#### UCLA-LS3-J

The UCLA-LS3-J version 3 [42] contains 20 items, with 4 choices per item: 1) never, 2) rarely, 3) sometimes, 4) always. Higher scores indicate a higher level of loneliness. This scale was selected because: 1) it is the only Japanese loneliness scale with established reliability and validity; 2) the version is recent; and 3) the English version and scale for different cultures has been used internationally [37–41], and therefore, comparisons can be made (Additional files 1 and 2).

#### Demographic data

Participants were asked for demographic information such as age, family structure, highest educational qualifications, employment status, economic status, and type of residence. Economic status was based on Japanese classification questions and answers about “subjective economic status” used in the national survey on a 4-point scale: 1) absolutely not affluent, 2) not affluent, 3) moderately affluent, and 4) affluent.

### External criteria

For external criteria used to evaluate validity, three variables were selected based on previous studies [35, 42]. These were subjective perceptions of health, feelings towards childrearing, and social network.

#### Subjective health perception

Subjective health perception was evaluated by having participants rate their perception of their current state of health on the following scale: 1) unhealthy. 2) not very healthy, 3) quite healthy, and 4) very healthy.

#### Feeling for childrearing

The feeling for childrearing scale [45, 46] was used. This scale comprises of 16 items in three domains: affirmative feeling for childrearing (four items), childrearing burden (six items), and childrearing anxiety (six items). Each item had four response options: 1) always, 2) sometimes, 3) rarely, and 4) never. The reliability and validity of this scale has been previously established [45, 46].

#### Social networks

The Japanese version of the Lubben Social Network Scale (LSNS-6 [47, 48]) was used to evaluate social networks. This scale was chosen because it allows for comparison of the relationship between the size and quality of social networks. The LSNS-6 comprises six items with six response options each evaluating the social networks of the mothers and their “family (3 items)” and “friends (3 items).” The reliability and validity of the Japanese version has been established [47, 48]. The total scores for this scale range from 0 to 30, with higher scores indicating a larger social network. A score of less than 12 marks the cutoff point for social isolation. The total scores for the two types of networks were calculated.

### Statistical analyses

#### Item analysis

##### Response distribution

The distribution of responses to all 20 items on the UCLA-LS3-J was calculated, and ceiling and floor effects and missing values were evaluated. The criteria for item analysis included ratings of response difficulty (missing data < 5.0%).

##### Good-poor analysis

To verify the discriminative power of each item, the difference in the mean values of the items in the first (top 25%) and fourth (bottom 25%) quartiles of the total scale scores were checked using a t-test.

##### Item-Total analysis

In order to verify the internal consistency of each item and of the scale, the correlation coefficient (Pearson product-moment correlation coefficient) of each item and the total scale scores, excluding the relevant item, were checked. Item-total correlations were determined using item-total correlations ≥0.70.

##### CFA

CFA was carried out in order to confirm whether the 20 items of the UCLA-LS3-J and of the SF-10 and SF-3 had the same single factor structure as the UCLA-LS3. The fit was evaluated using the goodness-of-fit index (GFI), adjusted goodness-of-fit index (AGFI), comparative fit index (CFI), and root mean square error of approximation (RMSEA). The criteria to accept the model was GFI, AGFI, and CFI values of ≥0.80, and RMSEA values of ≤0.10.

#### Reliability

The internal consistencies of the UCLA-LS3-J, SF-10, and SF-3 were evaluated using the Cronbach’s α coefficient. Factor reliability was considered acceptable if the Cronbach’s α was ≥0.70.

#### Validity review

Correlations between the demographic data and the external criterion, were correlated with the total scale scores of UCLA-LS3–3, SF-10, and SF-3. We analyzed if the answer to the three questions of the SF-3 would have been the same if the other 17 questions of the UCLA-LS3-J were not part of the questionnaire. Pearson’s correlation coefficient was used. IBM SPSS Amos 22 and SPSS version 22 statistical software (SPSS Inc., Chicago, IL, USA) were used to perform all statistical analyses.

## Results

### Respondent characteristics

Table 1 shows the participants’ characteristics. There were 248 valid responses. Of these, all respondents provided usable responses to all 20 items on the UCLA-LS3-J (usable response rate of 100%). The mean age was 32.7 ± 4.6 years. The most common family type was the nuclear family (89.5%) and 60.1% of participants were housewives. Almost all mothers perceived their health status as either “good health” (64.1%) or “moderately good health” (33.9%).

**Table 1:** Demographic data and external criteria

	*N* = 248	
	N (%) or Mean ± SD	
Demographic data		
Age (years)	32.7 ± 4.6	
Family structure		
Nuclear family	222	(89.5)
Extended family	20	(8.1)
Single-parent family	6	(2.4)
Occupational status		
Housewives	149	(60.1)
Office workers	69	(27.8)
Part-time workers	14	(5.6)
Self-employed	10	(4.0)
Students	3	(1.2)
Others	3	(1.2)
Educational status (highest educational qualifications)		
Junior high school graduate	5	(2.0)
High school graduate	59	(23.8)
Junior college graduate	81	(32.7)
University/graduate school graduate	102	(41.1)
Economic status		
Affluent	20	(8.1)
Moderately affluent	109	(44.0)
Not affluent	91	(36.7)
Absolutely not affluent	27	(10.9)
Type of residence		
Apartment	149	(60.1)
Independent housing	88	(35.5)
External criteria		
Social network (Lubbin Social Network Scale: LSNS-6)		
Family	7.1 ± 2.7	
Friends	6.3 ± 3.2	
Subjective Health Perception		
Good health	159	(64.1)
Moderately good health	84	(33.9)
Moderately poor health	5	(2.0)
Poor health	0	(0.0)
Feeling for childrearing scale		
Affirmative feeling for childrearing	13.6 ± 1.8	
Childrearing burden	12.7 ± 3.6	
Childrearing anxiety	12.3 ± 3.7	

Notes

Feeling for childrearing scale: The range of total scores for the “affirmative feeling for childrearing” is 4–16, while those for “childrearing burden” and “childrearing anxiety” are 6–24. Higher scores indicate a higher level for each feeling

Lubbin Social Network Scale: The total scores range from 0 to 30, with a higher score indicating a larger social network

### Item analysis on the UCLA-LS3-J

The scores obtained on the UCLA-LS3-J ranged from 20.0 to 73.0, with a mean of 38.4 ± 9.7, showing a normal distribution (Table 2). In the good-poor analysis, the first and fourth quartiles showed a significant difference on all items (*p* < 0.001). Furthermore, in the item-total analysis, the Pearson correlation coefficients for each item with the scores of the other 19 items were at least 0.30 (Table 2).

**Table 2:** Item analysis of the UCLA-LS3-J3 among mothers with infants and toddlers

							N = 248
Items	Mean	SD	Range	Median	Mode	Good-Poor analysis, p	I-T analysis, r
1	1.97	0.62	1.00–4.00	2.00	2.00	<0.001	.592**
2	2.42	0.87	1.00–4.00	3.00	3.00	<0.001	.590**
3	1.76	0.83	1.00–4.00	2.00	1.00	<0.001	.616**
4	1.79	0.82	1.00–4.00	2.00	1.00	<0.001	.679**
5	1.91	0.70	1.00–4.00	2.00	2.00	<0.001	.471**
6	2.17	0.66	1.00–4.00	2.00	2.00	<0.001	.526**
7	1.85	0.81	1.00–4.00	2.00	2.00	<0.001	.685**
8	2.03	0.74	1.00–4.00	2.00	2.00	<0.001	.575^**^
9	2.21	0.74	1.00–4.00	2.00	2.00	<0.001	.555**
10	1.68	0.66	1.00–4.00	2.00	2.00	<0.001	.667**
11	2.06	0.85	1.00–4.00	2.00	2.00	<0.001	.687**
12	1.53	0.71	1.00–4.00	1.00	1.00	<0.001	.529**
13	1.79	0.80	1.00–4.00	2.00	1.00	<0.001	.703**
14	1.84	0.82	1.00–4.00	2.00	1.00	<0.001	.781**
15	1.80	0.67	1.00–4.00	2.00	2.00	<0.001	.496**
16	1.81	0.77	1.00–4.00	2.00	2.00	<0.001	.570**
17	2.50	0.87	1.00–4.00	3.00	3.00	<0.001	.359**
18	2.23	0.76	1.00–4.00	2.00	2.00	<0.001	.681**
19	1.49	0.61	1.00–4.00	1.00	1.00	<0.001	.591**
20	1.52	0.62	1.00–4.00	1.00	1.00	<0.001	.661**
Total score	38.4	9.7	20.00–73.00	38.00	45.00		

**p < 0.01

### Reliability of the UCLA-LS3-J

The Cronbach’s α coefficient of the UCLA-LS3-J was 0.926, while those of the SF-10 and SF-3 were 0.888 and 0.790, respectively.

### CFA of the UCLA-LS3-J

The results of the CFA for the single-factor model indicated that the model’s goodness of fit was almost identical to that of the UCLA-LS3 and produced the following: UCLA-LS3-J (GFI = 0.882, AGFI = 0.840, CFI = 0.932, RMSEA = 0.066), SF-10 (GFI = 0.942, AGFI = 0.900, CFI = 0.956, RMSEA = 0.081), and SF-3 (GFI = 1.000, CFI = 1.000). The UCLA-LS3 was significantly correlated with the SF-10 (r = 0.965) and the SF-3 (r = 0.868). The SF3 was also significantly correlated with total score of the other 17 items of the UCLA-LS3 (r = 0.794).

### Validity of the UCLA-LS3-J

The relationships between the total score of the UCLA-LS3-J and demographic data, subjective health perception, feeling for childrearing scores, LSNS-6 scores were analyzed. In terms of total scores, significant negative correlations were found with subjective health perception (r = − 0.242, *p* < 0.001). Furthermore, a significant positive correlation was found with childrearing burden (r = 0.319, *p* < 0.001) and childrearing anxiety (r = 0.292, *p* < 0.001). However, a significant negative correlation was found with affirmative feeling for childrearing scores (r = − 0.294, *p* < 0.001) and all the LSNS-6 subscale scores (r = − 0.314 and − 0.438, *p* < 0.001) (Table 3).

**Table 3:** Relationship between the UCLA-LS3-J, SF-10, SF-3, and external criteria

						N = 248
	UCLA-LS3-J		SF-10		SF-3	
	r	p	r	p	r	p
Social network						
Family	−0.314	***	−0.280	***	−0.202	***
Friends	−0.438	***	−0.407	***	−0.348	***
Subjective Health Perception	−0.242	***	−0.222	***	−0.194	***
Feeling for childrearing						
Affirmative feeling for childrearing	− 0.294	***	− 0.287	***	− 0.174	***
Childrearing burden	0.319	***	0.310	***	0.237	***
Childrearing anxiety	0.292	***	0.310	***	0.255	***

***p < 0.001

r: Pearson’s coefficient of correlation

Notes: UCLA-LS3-J: Japanese version of the University of California, Los Angeles Loneliness Scale, 3rd version, SF: Short-form

Subjective health perception was evaluated on a scale of 4: 4) very healthy, 3) quite healthy, 2) not very healthy, 1) unhealthy

Feeling for childrearing scale: The range of total scores for the “affirmative feeling for childrearing” is 4–16, while those for “childrearing burden” and “childrearing anxiety” are 6–24. Higher scores indicate a higher level of each feeling

Lubbin Social Network Scale: The total scores range from 0 to 30, with a higher score indicating a larger social network

The SF-10 and SF-3 were significantly negatively correlated with subjective health perception (r = − 0.222 and − 0.194, *p* < 0.001) and affirmative feeling for childrearing scores (r = − 0.287 and − 0.174, *p *< 0.001). They were also significantly and positively correlated with childrearing anxiety (r = 0.310 and 0.255, *p* < 0.001) (Table 3).

## Discussion

This study aimed to verify the reliability and validity of the UCLA-LS3-J, and two short-form versions, for use with mothers with infants and toddlers. The characteristics of this scale are, as with the original, unidimensionality, not making use of the term “loneliness,” and evaluating loneliness in context. The widespread use of this scale is expected to lead to further empirical studies on loneliness in mothers with infants and toddlers. Additionally, because this scale is a 20-item, self-administered questionnaire, respondents can complete it quickly and easily. Using this scale would allow researchers to make individual- and population-level comparisons; thus this scale could be useful in the evaluation of loneliness in community health settings.

In terms of the reliability, the UCLA-LS3-J, SF-10, and SF-3 have high internal consistency (Cronbach’s α coefficients of the UCLA-LS3-J, SF-10, and SF-3 were 0.926, 0.888, and 0.790, respectively). This is also supported by the results of the item-total analysis. In terms of the validity of the scale, the correlation between the demographic data and the UCLA-LS3-J is similar to what was found with the UCLA-LS3. Further, there were no significant differences with regard to age. Finally, there were also similar findings to the UCLA-LS3, in the correlation between the external criteria and the UCLA-LS3-J; a significant correlation was observed with subjective health perceptions, feeling for childrearing, and social networks.

A significant correlation was found between the LSNS-6 and each subscale. This is consistent with Russell’s findings [35], and the findings of a study with Japanese elderly [42]. Specifically, loneliness had a weak but significant negative correlation with social relationships, such as the frequency of interactions with family and non-family members. No correlation was found with network density. The loneliness of mothers would be influenced by difference in quality, namely of interpersonal relationships and exchanges.

Finally, the mean score on the UCLA-LS3-J in this study was 38.4 ± 9.7 points. A similar result was found using the UCLA-LS3 with nurses in the United States, who scored 40.1 ± 9.5 points [35]. From this, it is possible to conclude that the loneliness of mothers with infants and toddlers in this study is similar to that of other adults, regardless of cultural background. Based on this, empirical studies with mothers and adult women using the UCLA-LS3-J could be conducted in the future in order to contribute to a more international discussion.

This study has several important findings. It used mothers as participants, in contrast to previous studies that included older adults and college students. These findings support the use of the UCLA-LS3-J and the SF-10 as measures in empirical studies that focus on loneliness and specifically, that study loneliness in mothers with infants and toddlers in the community. These results can also be used to inform programming for mothers with infants and toddlers to prevent and alleviate loneliness. Further, the SF-10 and SF-3 could be utilized as assessment tools when interviewing mothers, because they are simple questionnaires with high reliability and validity. In Japan, the local government can contact almost all mothers and infants through the national maternal health system because of the mandatory health check-ups for children. Interview sheets are used by public health nurses to interview mothers about their children’s health status and the mothers’ feelings of burden or anxiety about childrearing. However, the interview sheets have not been standardized across the various local governments.

There are several limitations to the present study. First, this study did not evaluate the test-retest reliability or discriminant validity. Further research is needed to clarify these aspects of the validity and reliability of the UCLA-LS3-J, SF-10, and SF-3. Second, the respondents in this study were all from a city in a metropolitan area of Japan. It would be beneficial to explore the psychometric properties of these scales in a more diverse population, such as mothers in other communities and those who at a particularly high risk of loneliness. Finally, the response rate was relatively low, although it was higher than that of previous studies targeted to mothers in Japan, which had a 30–40% response rate. The reason for the low response rate is that women had to mail in the responses, but had less time to do so because of busy parenting, work, and housework.

## Conclusion

The reliability and validity of the UCLA-LS3-J3 was explored in a study evaluating the feeling of loneliness of mothers with infants and toddlers. The SF-10, as a research measure for use in mothers with an infant and toddlers in Japan, was found to be a suitable alternative to the 20-item UCLA-LS3-J. The SF-10 and SF-3 are applicable to assessment of mothers with infants and toddlers in public health practice.

## Additional files

Additional file 1: The University of California, Los Angeles Loneliness Scale version 3 (UCLA-LS3) and two short-form versions—the 10-item UCLA-LS3(SF-10) and the 3-item UCLA-LS3(SF-3). (PDF 21 kb)

Additional file 2: A Japanese version of The University of California, Los Angeles Loneliness Scale version 3 (UCLA-LS3) and two short-form versions—the 10-item UCLA-LS3 (SF-10) and the 3-item UCLA-LS3 (SF-3). (PDF 17 kb)

## Data Availability

The datasets generated and analyzed during the current study are not publicly available because the Ethical Guidelines for Epidemiological Research by the Japanese Government, and the National Basic Resident Registration System administered by the Ministry of Internal Affairs and Communications in Japan, prohibit researchers from providing their research data to third-party individuals.

## Abbreviations

AGFI: Adjusted goodness-of-fit index
CFA: Confirmatory factor analysis
CFI: Comparative fit index
GFI: Goodness-of-fit index
RMSEA: Root mean square error of approximation
SF-10: Ten-item version of the University of California Los Angeles Loneliness Scale version 3
SF-3: Three-item version of the revised Short-form of University of California Los Angeles Loneliness Scale
UCLA-LS3: University of California Los Angeles Loneliness Scale version 3
UCLA-LS3-J: Japanese version of the University of California Los Angeles Loneliness Scale version 3

## References

[1] Perlman D, Peplau LA, Gilmour R, Duck S (1981). Toward a social psychology of loneliness. Personal relationships in disorder.

[2] Berguno G, Leroux P, McAinsh K, Shaikh S. Children’s experience of loneliness at school and its relation to bullying and the quality of teacher interventions. Qual Rep. 2004;9(3):483–99 https://nsuworks.nova.edu/tqr/vol9/iss3/7.

[3] Pinquart Martin, Sorensen Silvia (2001). Influences on Loneliness in Older Adults: A Meta-Analysis. Basic and Applied Social Psychology.

[4] Weeks David J. (1994). A review of loneliness concepts, with particular reference to old age. International Journal of Geriatric Psychiatry.

[5] Leigh-Hunt N., Bagguley D., Bash K., Turner V., Turnbull S., Valtorta N., Caan W. (2017). An overview of systematic reviews on the public health consequences of social isolation and loneliness. Public Health.

[6] Heinrich Liesl M., Gullone Eleonora (2006). The clinical significance of loneliness: A literature review. Clinical Psychology Review.

[7] Theeke Laurie A. (2009). Predictors of Loneliness in U.S. Adults Over Age Sixty-Five. Archives of Psychiatric Nursing.

[8] Jones Angela C., Schinka Katherine C., van Dulmen Manfred H. M., Bossarte Robert M., Swahn Monica H. (2011). Changes in Loneliness during Middle Childhood Predict Risk for Adolescent Suicidality Indirectly through Mental Health Problems. Journal of Clinical Child & Adolescent Psychology.

[9] Rew Lynn (2002). Relationships of Sexual Abuse, Connectedness, and Loneliness to Perceived Well-Being in Homeless Youth. Journal for Specialists in Pediatric Nursing.

[10] Kudo T, Nishikawa M. A study of the feeling of loneliness (1): The reliability and validity of the revised UCLA Loneliness Scale. 1983;22(2):99–108.

[11] Cacioppo John T., Hawkley Louise C., Thisted Ronald A. (2010). Perceived social isolation makes me sad: 5-year cross-lagged analyses of loneliness and depressive symptomatology in the Chicago Health, Aging, and Social Relations Study. Psychology and Aging.

[12] Liu Li-Juan, Guo Qiang (2008). Life satisfaction in a sample of empty-nest elderly: a survey in the rural area of a mountainous county in China. Quality of Life Research.

[13] Tijhuis M. (1999). Changes in and factors related to loneliness in older men. The Zutphen Elderly Study. Age and Ageing.

[14] Sorkin Dara, Rook Karen S., Lu John L. (2002). Loneliness, lack of emotional support, lack of companionship, and the likelihood of having a heart condition in an elderly sample. Annals of Behavioral Medicine.

[15] Elovainio Marko, Hakulinen Christian, Pulkki-Råback Laura, Virtanen Marianna, Josefsson Kim, Jokela Markus, Vahtera Jussi, Kivimäki Mika (2017). Contribution of risk factors to excess mortality in isolated and lonely individuals: an analysis of data from the UK Biobank cohort study. The Lancet Public Health.

[16] Bekhet Abir K., Zauszniewski Jaclene A., Nakhla Wagdy E. (2008). Loneliness: A Concept Analysis. Nursing Forum.

[17] Junttila Niina, Ahlqvist-Björkroth Sari, Aromaa Minna, Rautava Päivi, Piha Jorma, Räihä Hannele (2015). Intercorrelations and developmental pathways of mothers' and fathers' loneliness during pregnancy, infancy and toddlerhood - STEPS study. Scandinavian Journal of Psychology.

[18] Lubben James E. (1988). Assessing social networks among elderly populations. Family & Community Health.

[19] Valtorta NK, Kanaan M, Gilbody S, Hanratty B (2016). Loneliness, social isolation and social relationships: what are we measuring? A novel framework for classifying and comparing tools. BMJ Open.

[20] Dennis C-L, Hodnett E, Kenton L, Weston J, Zupancic J, Stewart D E, Kiss A (2009). Effect of peer support on prevention of postnatal depression among high risk women: multisite randomised controlled trial. BMJ.

[21] Sato M, Tadaka E, Arimoto A. Factors associated with loneliness among mothers with 4-month-old or 18-month-old infants in an urban area in Japan. Jpn J Public Health. 2014; 10.11236/jph.61.3_121. 10.11236/jph.61.3_121 24739939

[22] Baba C, Murayama H, Taguchi A, Murashima S. Loneliness and social relations among mothers with infants. Jpn J Public Health. 2013;10.11236/jph.60.12_727. 24429734

[23] Goto Aya, Quang Vinh Nguyen, Thi Tu Van Nguyen, Huu Phuc Trinh, Nghiem Minh Pham, Yabe Junko, Yasumura Seiji (2007). Maternal Confidence in Child Rearing: Comparing Data from Short-term Prospective Surveys Among Japanese and Vietnamese Mothers. Maternal and Child Health Journal.

[24] Masi Christopher M., Chen Hsi-Yuan, Hawkley Louise C., Cacioppo John T. (2010). A Meta-Analysis of Interventions to Reduce Loneliness. Personality and Social Psychology Review.

[25] Peplau LA, Perlman D, Peplau LA, Perlman D (1982). Perspectives on loneliness. Loneliness: a sourcebook of current theory, research and therapy.

[26] Kato Y [translation], Peplau LA, Perlman D. Kodokukan no Shinrigaku [Loneliness: A sourcebook of current theory, research, and therapy]. Tokyo, Japan: Seishin Shobou; 1988. p. 4–8.

[27] Florian Victor, Krulik Tamar (1991). Loneliness and social support of mothers of chronically ill children. Social Science & Medicine.

[28] Rokach Ami (2007). Self-Perception of the Antecedents of Loneliness among New Mothers and Pregnant Women. Psychological Reports.

[29] Strange Cecily, Fisher Colleen, Howat Peter, Wood Lisa (2014). Fostering supportive community connections through mothers' groups and playgroups. Journal of Advanced Nursing.

[30] Strange Cecily, Bremner Alexandra, Fisher Colleen, Howat Peter, Wood Lisa (2015). Mothers’ group participation: associations with social capital, social support and mental well-being. Journal of Advanced Nursing.

[31] Lee K, Vasileiou K, Barnett J. ‘Lonely within the mother’: an exploratory study of first-time mothers’ experiences of loneliness. Health Psychol. 2017; 10.1177/1359105317723451. 10.1177/1359105317723451 28795604

[32] Mandai M, Kaso M, Takahashi Y, Nakayama T. Loneliness among mothers raising children under the age of 3 years and predictors with special reference to the use of SNS: a community-based cross-sectional study. BMC Womens Health. 2018; 10.1186/s12905-018-0625-x.7. 10.1186/s12905-018-0625-x PMC6094879 30111371

[33] Geller JS (2004). Loneliness and pregnancy in an urban Latino community: associations with maternal age and unscheduled hospital utilization. J Psychosom Obstet Gynaecol.

[34] Hudson DB, Elek SM, Campbell-Grossman C (2000). Depression, self-esteem, loneliness, and social support among adolescent mothers participating in the new parents project. Adolescence..

[35] Russell Daniel W. (1996). UCLA Loneliness Scale (Version 3): Reliability, Validity, and Factor Structure. Journal of Personality Assessment.

[36] Russell Dan, Peplau Letitia Anne, Ferguson Mary Lund (1978). Developing a Measure of Loneliness. Journal of Personality Assessment.

[37] Elphinstone Brad (2017). Identification of a Suitable Short-form of the UCLA-Loneliness Scale. Australian Psychologist.

[38] Durak Mithat, Senol-Durak Emre (2010). Psychometric Qualities of the UCLA Loneliness Scale-Version 3 as Applied in a Turkish Culture. Educational Gerontology.

[39] Shevlin Mark, Murphy Siobhan, Murphy Jamie (2014). The Latent Structure of Loneliness. Assessment.

[40] Zarei Sahar, Memari Amir Hossein, Moshayedi Pouria, Shayestehfar Monir (2015). Validity and reliability of the UCLA loneliness scale version 3 in Farsi. Educational Gerontology.

[41] Boffo M, Mannarini S, Munari C. Exploratory structure equation modeling of the UCLA loneliness scale: a contribution to the Italian adaptation. TPM Test Psychom Methodol Appl Psychol. 2012. 10.4473/TPM19.4.7.

[42] Masuda Y, Tadaka E, Dai Y. Reliability and validity of the Japanese version of the UCLA Loneliness Scale Version 3 among the older population. Journal of Japan Academy of Community Health Nursing. 2012; 10.20746/jachn.15.1_25.

[43] Russell Dan, Peplau Letitia A., Cutrona Carolyn E. (1980). The revised UCLA Loneliness Scale: Concurrent and discriminant validity evidence. Journal of Personality and Social Psychology.

[44] Hughes Mary Elizabeth, Waite Linda J., Hawkley Louise C., Cacioppo John T. (2004). A Short Scale for Measuring Loneliness in Large Surveys. Research on Aging.

[45] Aramaki M (2005). The relationships among child-rearing anxiety, affirmative feeling for child-rearing, and social support: a comparison between single and married mothers. J Child Health.

[46] Aramaki M, Muto T. Factors related to negative and positive feelings about child-rearing: a survey of mothers of young children. Jpn J Dev Psychol. 2008; 10.11201/jjdp.19.87.

[47] Kurimoto Ayumi, Awata Shuichi, Ohkubo Takayoshi, Tsubota-Utsugi Megumi, Asayama Kei, Takahashi Kouko, Suenaga Katsuko, Satoh Hiroshi, Imai Yutaka (2011). Reliability and validity of the Japanese version of the abbreviated Lubben Social Network Scale. Nippon Ronen Igakkai Zasshi. Japanese Journal of Geriatrics.

[48] Lubben James, Blozik Eva, Gillmann Gerhard, Iliffe Steve, von Renteln Kruse Wolfgang, Beck John C., Stuck Andreas E. (2006). Performance of an Abbreviated Version of the Lubben Social Network Scale Among Three European Community-Dwelling Older Adult Populations. The Gerontologist.

