# Supplementary material for: Reliability and validity of Japanese versions of the UCLA loneliness scale version 3 for use among mothers with infants and toddlers: a cross-sectional study
Source: BMC Womens Health. 2019 Jul 26;19:105. doi: 10.1186/s12905-019-0792-4 (PMC6660924; doi:10.1186/s12905-019-0792-4)
Supplement: Supplementary file 1 — The University of California, Los Angeles Loneliness Scale version 3 (UCLA-LS3) and two short-form versions—the 10-item UCLA-LS3(SF-10) and the 3-item UCLA-LS3(SF-3). (PDF 21 kb) [file 12905_2019_792_MOESM1_ESM.pdf]

Supplementary file1

The University of California, Los Angeles Loneliness Scale version 3 (UCLA-LS3)[35] and two short-form versions—the 10-item UCLA-LS3 (SF-10)[35] and the 3-item UCLA-LS3 (SF-3)[44]

The following statements describe how people sometimes feel. For each statement, please indicate how you feel the way described by circling one number of each.	UCLA-LS3[35]	SF-10[35]	SF-3[44]	Never	Rarely	Sometimes	Always
1. *How often do you feel that you are “in tune” with the people around you?	●			1	2	3	4
2. How often do you feel that you lack companionship?	●	●	●	1	2	3	4
3. How often do you feel that there is no one you can turn to?	●			1	2	3	4
4. How often do you feel alone?	●			1	2	3	4
5. *How often do you feel part of a group of friend?	●			1	2	3	4
6. *How often do you feel that you have a lot in common with the people around you?	●	●		1	2	3	4
7. How often do you feel that you are no longer close to anyone?	●			1	2	3	4
8. How often do you feel that your interests and ideas are not shared by those around you?	●			1	2	3	4
9. *How often do you feel outgoing and friendly?	●			1	2	3	4
10. *How often do you feel close to people?	●	●		1	2	3	4
11. How often do you feel left out?	●	●	●	1	2	3	4
12. How often do you feel that your relationships with others are not meaningful?	●			1	2	3	4
13. How often do you feel that no one really knows you well?	●	●		1	2	3	4
14. How often do you feel isolated from others?	●	●	●	1	2	3	4
15. *How often do you feel you can find companionship when you want it?	●			1	2	3	4
16. *How often do you feel that there are people who really understand you?	●	●		1	2	3	4
17. How often do you feel shy?	●			1	2	3	4
18. How often do you feel that people are around you but not with you?	●	●		1	2	3	4
19. *How often do you feel that there are people you can talk to?	●	●		1	2	3	4
20. *How often do you feel that there are people you can turn to?	●	●		1	2	3	4

Scoring: Items (no.1, 5, 6, 9, 10, 15, 16, 19, 20) asterisked(*) should be reversed（i.e., 1＝4, 2＝3, 3＝2, 4＝1）, and the scores for each item then summed together. Higher scores indicate greater degrees of loneliness.

Reference:

35. Russell DW. UCLA Loneliness Scale (Version 3): reliability, validity, and factor structure. J Pers Assess. 1996; https://doi.org/10.1207/s15327752jpa6601_2
44. Hughes ME, Waite LJ, Hawkley LC, Cacioppo JT. A short scale for measuring loneliness in large surveys results from two population-based studies. Res Aging. 2004; http://dx.doi.org/10.1177/0164027504268574.
