# Supplementary material for: Reliability and validity of Japanese versions of the UCLA loneliness scale version 3 for use among mothers with infants and toddlers: a cross-sectional study
Source: BMC Womens Health. 2019 Jul 26;19:105. doi: 10.1186/s12905-019-0792-4 (PMC6660924; doi:10.1186/s12905-019-0792-4)
Supplement: Supplementary file 2 — A Japanese version of The University of California, Los Angeles Loneliness Scale version 3 (UCLA-LS3) and two short-form versions—the 10-item UCLA-LS3 (SF-10) and the 3-item UCLA-LS3 (SF-3). (PDF 17 kb) [file 12905_2019_792_MOESM2_ESM.pdf]

Supplementary file2

A Japanese version of The University of California, Los Angeles Loneliness Scale version 3 [35,42] and two short-form versions— the 10-item UCLA-LS3 (SF-10)[35] and the 3-item UCLA-LS3 (SF-3)[44]

日本語版 UCLA 孤独感尺度(第 3 版)，短縮版 10 項目,短縮版 3 項目

それぞれの項目について、あなたはどのくらいの頻度で感じているかお答えください。あてはまる番号ひとつに○をつけてください。	UCLA 孤独感尺度 第 3 版 [35,42]	10 項目 SF-10[35]	3 項目 SF-3[44]	決してない	ほとんどない	時々ある	常にある
1) 自分は周りの人たちの中になじんでいると感じますか*	●			1	2	3	4
2) 自分には人との付き合いがないと感じることがありますか	●	●	●	1	2	3	4
3) 自分には頼れる人が誰もいないと感じることがありますか	●			1	2	3	4
4)自分はひとりぼっちだと感じることがありますか	●			1	2	3	4
5) 自分は友人や仲間のグループの一員だと感じることがありますか*	●			1	2	3	4
6) 自分は周りの人たちと共通点が多いと感じることがありますか*	●	●		1	2	3	4
7) 自分は誰とも親しくしていないと感じることはありますか	●			1	2	3	4
8) 自分の関心や考えは周りの人たちにはわからないと感じることがありますか	●			1	2	3	4
9) 自分を社交的で親しみやすいと感じますか	●			1	2	3	4
10) 自分には親しい人たちがいると感じますか*	●	●		1	2	3	4
11) 自分は取り残されていると感じることがありますか	●	●	●	1	2	3	4
12) 他人との関わりは意味がないと感じることがありますか	●			1	2	3	4
13) 自分のことを本当によく知っている人は誰もいないと感じることはありますか	●	●		1	2	3	4
14) 自分は他の人たちから孤立していると感じることはありますか	●	●	●	1	2	3	4
15) 希望すれば自分と気の合う仲間は見つかると感じますか*	●			1	2	3	4
16) 自分を本当に理解している人がいると感じますか*	●	●		1	2	3	4
17) 自分は内気であると感じますか	●			1	2	3	4
18) 周りの人たちと一体感がもてないと感じることがありますか	●	●		1	2	3	4
19) 話し相手がいると感じますか*	●	●		1	2	3	4
20) 頼れる人がいると感じますか*	●	●		1	2	3	4

注)1, 5, 6, 9, 10, 15, 16, 19, 20 (*)は逆転項目(評定は 1＝4, 2＝3, 3＝2, 4＝1 に換算)

Reference:

35. Russell DW. UCLA Loneliness Scale (Version 3): reliability, validity, and factor structure. J Pers Assess. 1996; https://doi.org/10.1207/s15327752jpa6601_2

42.Masuda Y, Tadaka E, Dai Y. Reliability and validity of the Japanese version of the UCLA Loneliness Scale Version 3 among the older population. Journal of Japan Academy of Community Health Nursing. 2012; doi: 10.20746/jachn.15.1_25

44. Hughes ME, Waite LJ, Hawkley LC, Cacioppo JT. A short scale for measuring loneliness in large surveys results from two population-based studies. Res Aging. 2004; doi: 10.1177/0164027504268574.
